# Epidemiology and Antifungal Susceptibility Patterns of Invasive Fungal Infections (IFIs) in India: A Prospective Observational Study

**DOI:** 10.3390/jof8010033

**Published:** 2021-12-30

**Authors:** Yubhisha Dabas, Immaculata Xess, Mragnayani Pandey, Jaweed Ahmed, Janya Sachdev, Azka Iram, Gagandeep Singh, Manoranjan Mahapatra, Rachna Seth, Sameer Bakhshi, Rakesh Kumar, Viveka P. Jyotsna, Sandeep Mathur

**Affiliations:** 1 Department of Microbiology, All India Institute of Medical Sciences, New Delhi 110029, India; yubhi.aiims@gmail.com (Y.D.); miggipandey@gmail.com (M.P.); javidamd112@gmail.com (J.A.); drjanyasachdev@gmail.com (J.S.); fineiramkhan@gmail.com (A.I.); drgagandeep@gmail.com (G.S.); 2 Department of Hematology, All India Institute of Medical Sciences, New Delhi 110029, India; mrmahapatra@hotmail.com; 3 Department of Paediatrics, All India Institute of Medical Sciences, New Delhi 110029, India; drrachnaseth@yahoo.co.in; 4 Department of Medical Oncology, All India Institute of Medical Sciences, New Delhi 110029, India; sambakh@hotmail.com; 5 Department of Otorhinolaryngology, All India Institute of Medical Sciences, New Delhi 110029, India; winirk@hotmail.com; 6 Department of Endocrinology, All India Institute of Medical Sciences, New Delhi 110029, India; vivekapjyotsna@yahoo.com; 7 Department of Pathology, All India Institute of Medical Sciences, New Delhi 110029, India; mathuraiims@yahoo.com

**Keywords:** invasive fungal infections, invasive candidiasis, cryptococcosis, invasive aspergillosis, mucormycosis, risk factor, mortality, antifungal susceptibility

## Abstract

The epidemiology of invasive fungal infections (IFI) is ever evolving. The aim of the present study was to analyze the clinical, microbiological, susceptibility, and outcome data of IFI in Indian patients to identify determinants of infection and 30-day mortality. Proven and probable/putative IFI (defined according to modified European Organization for Research and Treatment of Cancer/Mycoses Study Group and AspICU criteria) from April 2017 to December 2018 were evaluated in a prospective observational study. All recruited patients were antifungal naïve (*n* = 3300). There were 253 episodes of IFI (7.6%) with 134 (52.9%) proven and 119 (47%) probable/putative infections. There were four major clusters of infection: invasive candidiasis (IC) (*n* = 53, 20.9%), cryptococcosis (*n* = 34, 13.4%), invasive aspergillosis (IA) (*n* = 103, 40.7%), and mucormycosis (*n* = 62, 24.5%). The significant risk factors were high particulate efficiency air (HEPA) room admission, ICU admission, prolonged exposure to corticosteroids, diabetes mellitus, chronic liver disease (CLD), acquired immunodeficiency syndrome (AIDS), coronary arterial disease (CAD), trauma, and multiorgan involvement (*p* < 0.5; odds ratio: >1). The all-cause 30-day mortality was 43.4% (*n* = 110). It varied by fungal group: 52.8% (28/53) in IC, 58.8% (20/34) in cryptococcosis, 39.8% (41/103) in IA, and 33.9% (21/62) in mucormycosis. HEPA room, ICU admission for IC; HEPA rooms, diabetes mellitus for cryptococcosis; hematological malignancies, chronic kidney disease (CKD), sepsis, galactomannan antigen index value ≥1 for IA and nodules; and ground glass opacities on radiology for mucormycosis were significant predictors of death (odds ratio >1). High minimum inhibitory concentration (MIC) values for azoles were observed in *C. albicans*, *C. parapsilosis*, *C. glabrata*, *A. fumigatus*, *A. flavus*, *R. arrhizus*, *R. microsporus*, and *M. circinelloides*. For echinocandin, high MIC values were seen in *C. tropicalis*, *C. guillermondii*, *C. glabrata*, and *A. fumigatus*. This study highlights the shift in epidemiology and also raises concern of high MICs to azoles among our isolates. It warrants regular surveillance, which can provide the local clinically correlated microbiological data to clinicians and which might aid in guiding patient treatment.

## 1. Introduction

Invasive fungal infections (IFIs) continue to represent a significant problem in immunocompromised individuals and a large proportion of critically ill patients [1]. However, this changing epidemiology with increasing numbers of immunocompetent hosts includes the cases following natural disasters and large iatrogenic inoculation [1,2]. On-going pandemic caused by severe acute respiratory syndrome coronavirus 2 (SARS-CoV-2) has also brought the focus back on superinfections caused by secondary IFIs [3].

Over the past few decades, incidence of IFIs has also been increasing. This is attributed primarily to the overall increase in the number of patients irrespective of severe immunosuppression such as acquired immunodeficiency syndrome (AIDS), hematological malignancies, organ transplantation, etc. or apparent immunocompetent status with diabetes mellitus, chronic obstructive pulmonary disease (COPD), etc. [1,4]. Depending upon the population cohorts, the overall IFI incidence rate varies from 3% to 20% [5,6,7,8,9,10,11]. Opportunistic pathogens such as *Candida* sp., *Cryptococcus* sp., *Aspergillus* sp., and Mucorales are the most common causative agents of these infections. There are other hyalohyphomycetes such as *Fusarium* sp. and *Scedosporium* sp., phaeohyphomycetes (darkly pigmented or dematiaceous fungi), and basidiomycetous yeasts (*Trichosporon* sp., *Malassezia* sp.) known to cause these infections in different populations [12]. These fungi affect various tissues throughout the body, with the respiratory system being the most common [13]. Invasive candidiasis is considered the most common IFI; however, there are shifts in epidemiology noted towards non-albicans sp. [4,14]. In hematological diseases, a predominance of invasive aspergillosis (IA) has been reported [1,4]. In critically ill patients, these infections can also present as coinfections, further complicating and delaying the diagnosis [13].

In any economic scenario, the most feasible IFI diagnostic modality—fungal culture and pathological examination—is not conducive, as it does not meet the urgent diagnosis requirement and thereby delays treatment, resulting in a high fatality rate [13]. This is further complicated by the increased occurrence of resistant species owing to a surge in antifungal prophylaxis and emergence of previously rare fungal species displaying inherent resistance to common antifungal agents used [4,15,16]. A key determinant for the outcome of IFIs is early initiation of antifungal therapy [17]. There are established guidelines for the four commonest IFIs—invasive candidiasis (IC), Cryptococcosis, invasive aspergillosis (IA), and mucormycosis—from the Infectious Diseases Society of America (IDSA), the European Society for Clinical Microbiology and Infectious Diseases, and the European Confederation of Medical Mycology (ESCMID/ECMM) [18,19,20,21]. However, uncertainty lingers about the interpretation of antifungal susceptibility testing (AST) and the significance of minimal inhibitory concentration (MIC) in predicting outcome [17]. Regardless, IFIs are a major cause of morbidity and mortality [4]. Careful consideration of local fungal epidemiology describing clinical characteristics, prognostic factors, use of diagnostic algorithms and antifungal susceptibility patterns can prove useful for overcoming these shortcomings [1].

However, there are a limited number of studies from India, which renders many aspects of IFI poorly understood. This lacuna in data prompted us to conduct this study aimed at analyzing clinical, microbiological, susceptibility, and outcome data of IFIs to support clinicians when deciding on prophylactic or empirical antifungal therapy.

## 2. Materials and Methods

### 2.1. Study Design and Data Collection

This was a prospective observational study to investigate IFI epidemiology from April 2017 to December 2018 conducted at the Department of Microbiology in collaboration with Departments of Hematology, Medical Oncology, Pediatrics, Sleep Disorders and Pulmonary Medicine, Otorhinolaryngology, Endocrinology, Medicine and Pathology at a tertiary care hospital, All India Institute of Medical Sciences, New Delhi, India.

Patients clinically suspected of IFI displaying at least one of the following host factors were enrolled in the study: hematologic malignancy; cancer and receiving chemotherapy within the last 3 months before admission, with or without neutropenia; chronic obstructive pulmonary disease (COPD); transplant recipient (hematopoietic/solid organ); chronic granulomatous disease (tuberculosis); other immunocompromised state (inherited immunodeficiency, child C cirrhosis, or HIV, etc.); steroid use—at least 4 mg methylprednisolone (or equivalent) a day for at least 7 days in the 3 weeks before admission or during the course of the ICU stay for at least 5 days or a cumulative dose of at least 250 mg of methylprednisolone (or equivalent) in the past 3 months before enrolment; recipient of any other immunosuppressive treatment (tacrolimus, cyclosporine, methotrexate, cyclophosphamide, etc.); diabetes mellitus with or without ketoacidosis; or microbiological evidence of *Aspergillus* infection during the stay in ICU (any positive culture or two positive circulating galactomannan tests) (data not shown). In addition, eligible patients could only be enrolled if they had at least two of the following three features: fever refractory to at least 3 days of appropriate antibiotics or fever relapsing after a period of defervescence of at least 48 h while still receiving antibiotics; clinical signs and/or symptoms suggestive of invasive mycosis: pleuritic chest pain or physical finding of pleural rub, or one of the following symptoms of lower respiratory tract infection (new sputum secretions, dyspnea, or hemoptysis); or development of new pulmonary infiltrates on chest X-ray. To enhance the homogeneity of the study population, only antifungal-naïve patients were included. The sole exclusion criterion was patients on antifungal prophylaxis or preexisting antifungal treatment. Baseline demographic, clinical characteristics, 30-day all-cause mortality details were recorded. Hospitalization data (general ward/high-efficiency particulate air (HEPA) units/intensive care units (ICU)) were also collected.

Ethics statement: The study was conducted according to the guidelines of the Declaration of Helsinki, and the study was approved by the ethics committee of the institute i.e., All India Institute of Medical Sciences, New Delhi, India (Ref no. IEC/NP-25/2014RP-10/2014, OP-3/09.02.2017). The detailed procedure was as per institute guidelines: http://www.aiims.edu/aiims/academic/ethics-committee/forms%20in%20pdf/IEC/Format_of_Institution_Ethics_Committee_15032012.pdf (accessed on 16 January 2017). The consent forms for minor/incapable participants were obtained by their LAR, i.e., legally accepted representatives (example: mother, father, children, or grandparents).

### 2.2. Definition of IFI

Three thousand three hundred consecutive patients who fulfilled European Organization for Research and Treatment of Cancer/Invasive Fungal Infections Cooperative Group and the National Institute of Allergy and Infectious Diseases Mycoses Study Group (EORTC/MSG) 2008 definitions for possible, probable, or proven IFI [22] and AspICU criteria for clinically suspected invasive aspergillosis (IA) in ICUs [23] were enrolled. However, for analysis, only the probable and proven IFIs were included, as per the new EORTC/MSG 2020 definitions [24].

### 2.3. Diagnosis of IFI

Samples were processed following conventional mycological procedures including direct microscopy (visualization of capsule on negative staining, budding yeast cell on grams stain, septate or aseptate hyphae on KOH mount) and growth on sabouraud dextrose agar and CHROMagar. The isolates were identified by microscopy (slide culture on Tween 80 corn meal agar, septate hyphae on lactophenol cotton blue mount, and aseptate hyphae on calcofluor mount) and morphology on CHROMagar, bird seed agar, malt extract agar, and urea hydrolysis. Galactomannan antigen (GM) assay was performed using Platelia kit (Bio-Rad, Marnes-la-Coquette, France). Serial serum samples (day 0 and day 7) were obtained for all the patients who were clinically suspected of IA for a uniform GM analysis as per the physician’s recommendation. Capsular antigen of *Cryptococcus* was detected using latex agglutination test (LAT) of Pastorex^TM^ Crypto Plus (Bio-Rad, Marnes-la-Coquette, France). Only the isolates difficult to speciate phenotypically were subjected to DNA sequencing, where segments of DNA comprising the ITS region were amplified with primers ITS1 (5′-TCCGTAGGTGAACCTGCGG-3′) and ITS4 (5′-TCCTCCGCTTATTGATATGC-3′). Invasive/sterile site samples such as cerebrospinal fluid (CSF), bronchoalveolar lavage (BAL), pleural fluid, and tissue biopsy were collected at the discretion of the attending physician, while considering the debilitated condition of thrombocytopenic patients.

### 2.4. Antifungal Susceptibility Patterns

Antifungal susceptibility testing was performed using the broth microdilution assay according to Clinical Laboratory Standards Institute (CLSI) approved standard M-60 for yeasts [25] and M-38 for molds [26]. Quality control isolates (*Candida parapsilosis* ATCC 22019, *Candida krusei* ATCC 6258, and *Aspergillus flavus* ATCC 204304) were included. All assays were done in duplicates. The antifungal drugs tested were: amphotericin B, flucytosine, itraconazole, fluconazole, voriconazole (Sigma Chemical Corporation, St. Louis, MO, USA); caspofungin, posaconazole (Pfizer Pharmaceuticals, New York, NY, USA); micafungin (Astellas Pharmaceuticals, Tokyo, Japan).

The inoculum suspensions for yeasts were prepared using 0.5 McFarland [25] and for molds conidial suspensions were prepared in RPMI 1640 and adjusted to final concentration of 2.5 × 10^4^ CFU/mL, as previously described [26]. The assays were incubated at 35 °C for 24/48 h except for *Cryptococcus* sp., where the incubation was extended for 72 h.

For *Candida* sp. the breakpoints followed were according to the M-60 CLSI document [25]. For *Cryptococcus* sp. and *Aspergillus* sp., breakpoints used were defined in previous studies from our laboratory [27,28]. For mucorales, break points referred by Almyroudis et al. were used for analysis, viz., amphotericin B ≤ 1 μg/mL, itraconazole ≤ 0.5 μg/mL, and posaconazole ≤ 0.5 μg/mL [29].

### 2.5. Statistical Analysis

Continuous variables are presented as either mean (±SD) or median, with interquartile range in case of skewed distribution. They were normally distributed and the Student’s t-test was used. The categorical variables are expressed as numbers and percentages of the group from which they were derived. The chi-square test and Fisher’s exact test were used to compare categorical variables as appropriate. Socio-demographic clinical characteristics and risk factors were evaluated by univariate and multivariate analysis. These were entered into a logistic regression model for calculation of unpaired and paired odds ratios (ORs). The ORs are given with 95% confidence intervals (CIs). A cutoff of *p* ≤ 0.05, two-tailed, was considered significant for all statistical analysis.

All statistical analysis was conducted using STATA version 9 (StataCorp. 2005. Stata Statistical Software: Release 9. College Station, TX: StataCorp LP) except for antifungal data which was statistically analyzed with Statistical Package for the Social Sciences software (version 16.0; SPSS S.L., Madrid, Spain).

## 3. Results

**Figure 1:**
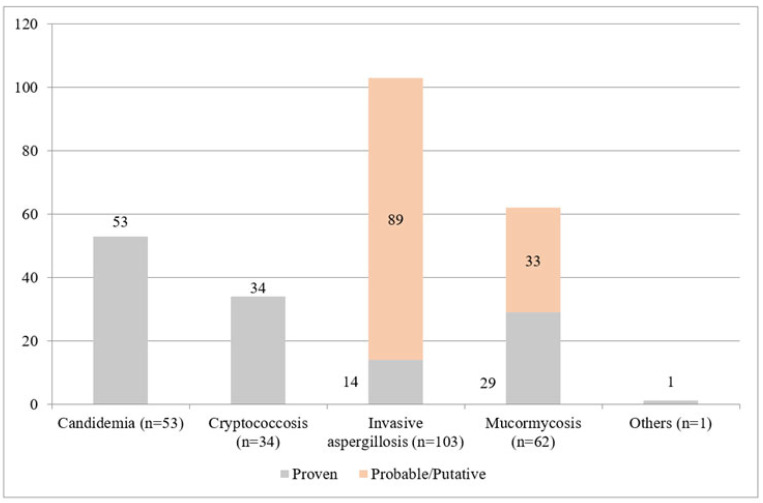
Cases distribution as proven and probable/putative IFI.

Three thousand and three hundred patients suspected of IFIs were recruited in the study, of which 253 (253/3300, 7.6%) (52%, 134/253 proven and 48%, 119/253 probable/putative IFIs) presented with 65.6%, 166/253 mold (invasive aspergillosis, mucormycosis, and one case of taleromycosis) and 34.4%, 87/253 yeast IFIs (invasive candidiasis and cryptococcosis). The case distribution of as proven and probable/putative IFIs is shown in Figure 1. The most common sites of involvement were lung (42.7%, 108/253) and bloodstream (20.9%, 53/253) (Table 1). Eighteen episodes of infection were diagnosed by direct microscopy alone. Culture positive infections (235/253, 92.9%) were caused by a wide range of fungal species (Supplementary Figure S1). Species domination was by *Candida albicans* and *Candida parapsilosis* (26.4% each, 14/53) in invasive candidiasis cases, by *Cryptococcus neoformans* (100%, 34/34) in cryptococcosis, by *Aspergillus fumigatus* (41.8%, 43/103) and *Aspergillus flavus* (40.8%, (42/103) in invasive Aspergillosis, and by *Rhizopus arrhizus* (48.4%, 30/62) in mucormycosis (Supplementary Figure S1).

The demographic and clinical characteristics describing statistically significant comorbidities and underlying conditions of the patients are listed in Table 2. The majority of subjects were male (66%, 167/253) and were hospitalized in open general ward (55.7%, 141/253). As shown in Figure 1, there were 53% (134/253) proven IFIs and 47% (119/ 253) probable/putative IFIs. High-efficiency particulate air (HEPA) room and ICU hospitalizations were found significantly associated with IFIs (odds ratio, OR > 1). On multivariate analysis, comorbidities such as long term corticosteroids, diabetes mellitus, acquired immunodeficiency syndrome (AIDS), chronic liver disease (CLD), coronary arterial disease (CAD), and trauma were found to be significant predictors of IFI (odds ratio > 1) (Table 2). Individual drugs of choice for definitive treatment were liposomal amphotericin B (30.4%, 77/253), followed by voriconazole (26.9%, 68/253). Overall 30-day mortality was 43.5% (110/253).

### 3.1. Invasive Candidiasis

**Figure 2:**
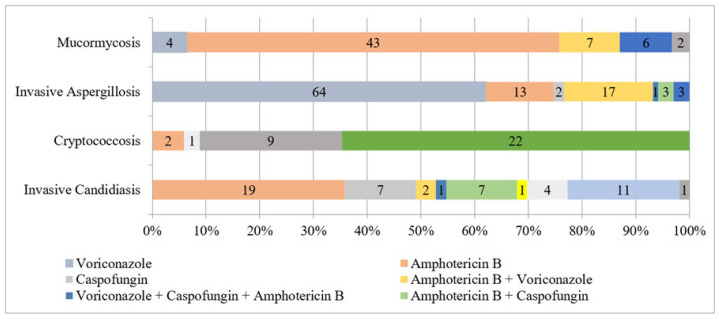
Antifungal treatment in IFI.

All *Candida* infections were bloodstream. Multiple drugs, single and combinational, were used for treatment with liposomal amphotericin B being used in 35% (19/53) of cases, followed by a combination of fluconazole with an echinocandin in 20.8% (11/53) of cases (Figure 2). Irrespective of the class of drug, the duration of treatment ranged from 3 to 28 days (median, 11 days; IQR, 7 days). Overall 30-day mortality was 52.8% (28/53). Isolation of *Candida tropicalis* (OR 4, 95% CI 0.6–26), *Candida parapsilosis* (OR 1, 95% CI 0.2–4.4), and *Candida pelliculosa* (OR 1, 95% CI 0.1–19) was associated with poor outcome. On univariate analysis, other variables that significantly predicted mortality were age (OR 1.01, 95% CI 0.98–1.03), sex (OR 1.42, 95% CI 0.47–4.24), HEPA room hospitalization (OR 2.5, 95% CI 0.5–12.5), ICU hospitalization (OR 1.7, 95% CI 0.5–5.8), chronic kidney disease (CKD) (OR 1.9, 95% CI 0.3–11.4), pulmonary manifestations (OR 1.14, 95% CI 0.26–4.8), and multiorgan involvement (OR 3.13, 95% CI 0.57–17.2).

### 3.2. Cryptococcosis

**Figure 3:**
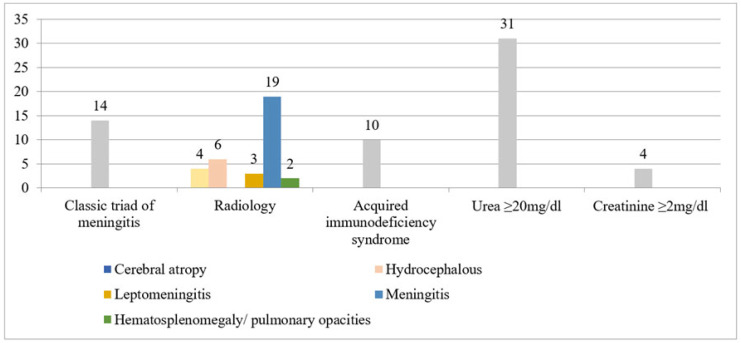
Clinical characteristics of Cryptococcosis.

Other than two pulmonary cases (5.6%, 2/34), all had cerebral presentation (94.4%, 32/34). A total of 24 (70.6%, 24/34) were culture positive with *Cryptococcus neoformans*, whereas 10 (29.4%) only showed budding round yeast cell with halo on india ink staining. Latex agglutination testing for the capsular antigen was carried out for all patients (data not shown). Clinical characteristics noted are shown in Figure 3. Flucytosine with L-amphotericin B was given in 64.7% (22/34) of cases, and fluconazole with L-amphotericin B was treatment of choice in 24.5% (9/34) of cases. Overall 30-day mortality was 58.8% (20/34). Patients were mostly admitted in open general wards 25/34 (73.5%), with only nine in HEPA room hospitalizations (26.5%), and admission in the latter was associated with poor outcome *p*= 0.19 (OR 3.23, 95% CI 0.55–18.7). On univariate analysis, significant predictors of mortality were hospitalization duration (OR 1.01, 95% CI 0.85–1.2), chronic granulomatous diseases (CGD) (OR 1.33, 95% CI 0.33–5.26), diabetes mellitus (OR 2.29, 95% CI 0.21–24.6), AIDS (OR 1.97, 95% CI 0.4–9.5), and CKD (OR 1, 95% CI 0.25–3.91).

### 3.3. Invasive Aspergillosis

**Figure 4:**
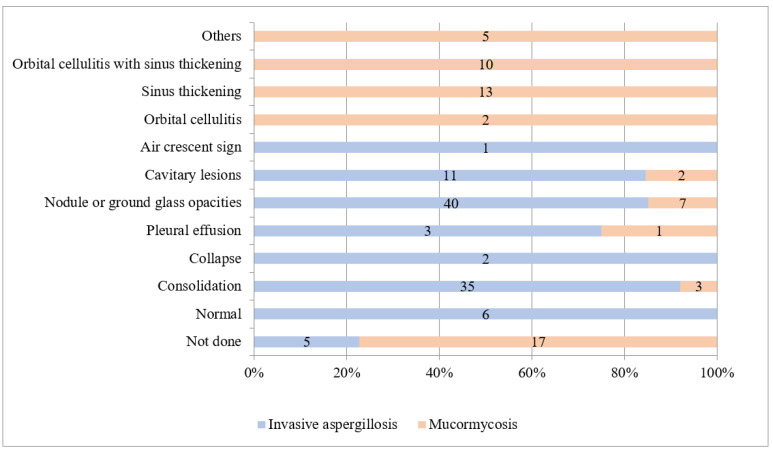
Radiological findings in invasive aspergillosis and mucormycosis cases.

The cases were mainly of invasive pulmonary aspergillosis (91.3%, 94/103). The treatment of choice was voriconazole in 61.5% (64/103) cases, followed by a combination of L-amphotericin B and voriconazole in 16.5% (17/103) cases. All were direct microscopy and culture positive. Single isolates from repeat isolations were included for respiratory non-invasive samples (data not shown). Galactomannan antigen testing was performed for all patients (data not shown). Radiological findings are shown in Figure 4. Overall 30-day mortality was 39.8% (41/103). Isolation of *Aspergillus fumigatus* (OR 1.9, 95% CI 0.8–4.6), *Aspergillus nidulans* (OR 1, 95% CI 0.08–12), and *Aspergillus niger* (OR 1.2, 95% CI 0.25–5.7) was associated with poor outcome. Administration of L-amphotericin B (OR 1.04, 95% CI 0.3–3.55) and a combination of the former with posaconazole (OR 3.33, 95% CI 0.28–38.7) was associated with poor outcome. Radiological picture suggestive of lung collapse (OR 1.5, 95% CI 0.05–40.6) and appearance of nodules and ground glass opacities (OR 1.5, 95% CI 0.22–9.96) also predicted mortality. On univariate analysis, other variables that significantly predicted mortality were age (OR 1.03, 95% CI 1.01–1.05), sex (OR 1.1, 95% CI 0.47–2.56), ICU hospitalization (OR 4.27, 95% CI 1.73–10.53), hematological malignancy (OR 2.48, 95% CI 1.07–5.73), CKD (OR 3.67, 95% CI 1.6–8.5), prolonged corticosteroids (OR 1.56, 95% CI 0.7–3.48), mechanical ventilation (OR 2.77, 95% CI 1.21–6.36), sepsis (OR 3.67, 95% CI 1.15–11.72), and high galactomannan antigen index value of ≥1 (OR 1.6, 95% CI 0.72–3.56).

### 3.4. Mucormycosis

**Figure 5:**
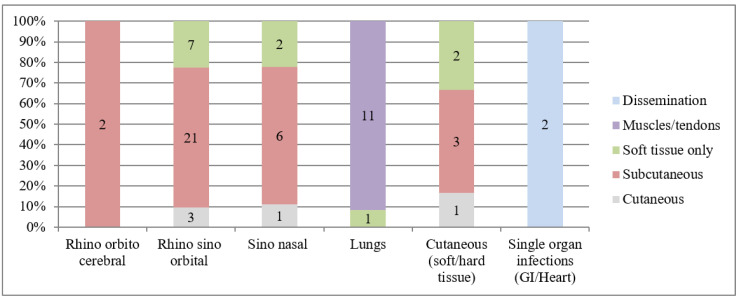
Site and tissue involvement in mucormycosis.

Fifty percent (31/62) of cases were rhino-orbital with sinus involvement. Overall site and tissue involvement is shown in Figure 5. All were direct microscopy positive with 87% (54/62) of culture growth. Surgical debridement was performed in 71% (44/62) of cases, with L-amphotericin B (43/62, 69%) as the most common antifungal used. Overall 30-day mortality was 33.9% (21/62). *Rhizopus microsporus* (OR 1.94, 95% CI 0.32–11.75) and *Mucor* isolations (OR 3.33, 95% CI 0.2–54.5) were associated with poor outcome. On radiology, appearance of consolidation (OR 1.2, 95% CI 0.08–16.4), nodules/ground glass opacities (OR 8.4, 95% CI 1.27–55.4), and sinus thickening (OR 2.05, 95% CI 0.45–9.3) were associated with poor outcome. On univariate analysis, other significant predictors of mortality were age (OR 3.33, 95% CI 0.2–54.5), hematological malignancy (OR 3.33, 95% CI 0.2–54.5), CKD (OR 3.33, 95% CI 0.2–54.5), symptoms duration (OR 3.33, 95% CI 0.2–54.5), pulmonary mucormycosis (OR 3.33, 95% CI 0.2–54.5), and ketoacidosis (OR 3.33, 95% CI 0.2–54.5). Multivariate analysis of mortality predictors for all IFIs is listed in Table 3.

### 3.5. Antifungal Susceptibility Testing

All experiments were performed in duplicates, and the MIC values of quality control strains fell within the established ranges published by CLSI methodologies. Table 4 summarizes the in vitro susceptibility value ranges, geometric mean, mode, MIC_50_, and MIC_90_ values of all the isolates to the antifungals tested. Irrespective of genera, all isolates were susceptible to amphotericin B. Based on breakpoints for different fungi, high MIC values (intermediate and resistant combined) were recorded for fluconazole in 3 *C. albicans*, 4 *C. parapsilosis*, and 1 *C. guillermondii*; for voriconazole in 3 *C. albicans*, 3 *C. parapsilosis*, and 5 *C. glabrata*; for itraconazole in 4 C. albicans, 4 *C. parapsilosis*, 3 *C. glabrata*, 1 *A. flavus*, 1 *A. fumigatus*, 1 *A. niger*, 12 *R. arrhizus*, 3 *R. microsporus*, and 1 *Mucor circinelloides*; for posaconazole in 15 *R. arrhizus*, and 7 *R. microsporus*; for caspofungin in 1 *C. albicans*, 2 *C. tropicalis*, 4 *C. glabrata*, and 2 *A. fumigatus*; and for micafungin in *3 C. tropicalis*, 2 *C. guillermondii*, and 4 *C. glabrate* (Table 5).

## 4. Discussion

We describe epidemiology, predisposing factors, antifungal susceptibility patterns, and outcome in invasive fungal infections (IFIs) from a tertiary care center in India. Overall, we found a significant incidence of IFIs in our mixed but high-risk cohort of patients. The observed rate of 7.6% in this study is in accordance with previously published literature of IFIs in antifungal-naïve population [5,6,7,8,9,10,11,30,31,32,33,34].

In our mixed cohort of patients, invasive aspergillosis (IA) (40.7%, 103/253) emerged as the most common IFI, followed by mucormycosis (24.5%, 62/253). This is in accordance with studies conducted in the hematological patient population [34,35] and in contrast with reports of invasive candidiasis being the most common IFI [13,36]. Traditional underlying conditions noted in this study were dominated by prolonged exposure to corticosteroids (24.5%), diabetes mellitus (23.7%), and hematological malignancies (19.8%). Among other predisposing conditions, chronic kidney disease (CKD) (34.4%) and pulmonary manifestations (30.8%) were the most prevalent. These data are consistent with the range of patient populations affected inside and outside of traditional high-risk groups [7,37,38,39,40,41,42,43]. Contrary to global data, 22.1% of IFI cases noted in this study were from the ICUs, with a predominance of 55.3% IA followed by 33.9% IC and the remaining 10.7% of mucormycosis cases [44,45].

In this study, species identification revealed that *Candida albicans* and *Candida parapsilosis* were the most common cause of invasive candidiasis (IC), which is incongruent with the listing of *Candida tropicalis* as the most common cause of IC from India [46,47]. However, fungal isolations of *Cryptococcus neoformans*, *Aspergillus flavus*, and *Rhizopus arrhizus* were in accordance with previously published literature on respective IFIs [27,48,49,50].

Similar to global data in our invasive candidiasis isolates, azole resistance was noted in *C. albicans*, *C. parapsilosis*, and *C. glabrata*, whereas echinocandin resistance was noted in *C. albicans* and *C. glabrata* [51,52,53,54,55]. It is significant that for echinocandins, in vitro susceptibility tested resistance is known to translate into treatment failures owing to FKS mutations [52,53,56]. Previous invasive candidiasis data from our center listed <6% resistance to fluconazole and 100% sensitivity to amphotericin B [57]. Global data support the CLSI *C. albicans* clinical breakpoints for fluconazole, whereas lacking the similar acceptance for *C. glabrata*, the most isolates of this non-albicans species fall in the intermediate category [17]. To overcome these shortcomings, susceptibility testing research has broadened. Natural oils from algae such as *Ruta graveolans* or north Sardinia plants have been evaluated for their efficacy (fungistatic and fungicidal). They have been found active against multidrug-resistant *Candida* sp. [58,59].

Although once known to be rare, cryptococcosis has occurred at a high frequency in India in the past two decades, as envisaged in a recent multicenter study [50]. It is one of the AIDS-defining infections and is responsible for about 15% of AIDS-related deaths [60]. However, in the current study, 50% of cases were seen with renal involvement, and only 29.4% were AIDS-related. The decrease in AIDS-related secondary cryptococcal infection may be owing to highly active antiretroviral therapy (HAART) therapy [60].

For Cryptococcosis, the drugs of choice are described in detail [19]. Amphotericin B (and its lipid formulations) with flucytosine is indicated as induction therapy in HIV-infected individuals, organ transplant recipients, non-HIV, and non-transplant patients, with differences in dosage and duration. The maintenance and consolidation therapy is fluconazole. For patients with CD4 count >100 cells/µL and undetectable viral load for >3 months, a minimum of 1 year of antifungal therapy is recommended [19]. From India, high MICs against fluconazole and flucytosine have been reported [61,62,63]. However, from our center in the current and another multicenter study [19], 100% sensitivity was noted for all the drugs.

In Western countries, local epidemiology highlights the predominance of *A. fumigatus* in invasive aspergillosis (IA) cases [64,65], whereas from India, *A.flavus* is most commonly isolated. Voriconazole is the drug of choice for primary therapy (especially with cases of invasive pulmonary aspergillosis) [20,66]. However, triazole (itraconazole, voriconazole, and posaconazole) drug resistance has been previously reported [64,67,68]. In this study, only three strains showed high MICs to itraconazole, of which one was *A. fumigatus*. In the Western world, *Aspergillus fumigatus* azole resistance (ARAF) has been extensively researched for its clinical implications [69,70,71,72], whereas from India, there are few sporadic reports of clinical and environmental ARAF strains [28,73,74,75].

Another life-threatening IFI that was noted in high numbers in this study was mucormycosis. It presented in its most common form, rhino-orbital, and with the usual predisposing conditions of renal involvement and ketoacidosis [48]. The increasing trends of this infection hint towards breakthrough infections [76,77]. Antifungal treatment strategies are generally associated with surgical intervention for these cases. The focus is on the roles of amphotericin B formulations, posaconazole, combination therapies, and newer therapeutic approaches [78]. It is important to identify the genus, or if possible the species, since *Cuninghamella*, *Lichtheimia*, and *Rhizopus* oryzae can be resistant to posaconazole, which usually shows susceptible MIC profiles [79,80]. The standard treatment is liposomal amphotericin B dose according to the localization and extent of infection. The role of posaconazole is that it can be used as salvage therapy along with amphotericin B [80,81]. Incongruent with amphotericin B susceptibility data from India, in this study all strains were susceptible [82,83,84]. However, about 70% of *Rhizopus* species were susceptible to posaconazole, which is similar to previously published data [82,83,84].

Novel antifungal therapies and strategies can aid in the management of IFIs. In high-risk patients (neutropenic, etc.), antifungal prophylaxis is also recommended. However, the benefits associated with antifungal therapy (prophylactic/empirical) need to be evaluated with respect to local epidemiology and cost effectiveness. The treatment modalities are still unavailable/unaffordable to many patients in a developing nation such as ours.

The study was limited by its clinical suspicion inclusion bias and unexpectedly low numbers of probable IFIs, which may be due to the lack of invasive sampling owing to the poor condition of patients. There was one *Taleromyces marneffi* recovered from an AIDS patient, limiting the overall picture of the burden of rare pathogens among these infections.

## 5. Conclusion

In conclusion, the local epidemiology of IFIs in this study was significantly different from elsewhere. The predictors of infection or mortality were found similar to global data. However, these considerations underscore the importance of understanding both the epidemiology and resistance profile of the invasive fungal isolates that are commonly seen in both immunocompromised and immunocompetent populations. An active surveillance of invasive fungal infections, along with multidrug susceptibility testing of isolates to monitor the extent of the problem and develop feasible local diagnostic algorithms, will provide the database that might aid in future treatments to limit the emergence of resistance and alleviate the fatality rate.

## Figures and Tables

**Table 1:** Site of invasive fungal infections (IFI).

Infection Site	Invasive Candidiasis (*n* = 53)	Cryptococcosis (*n* = 34)	Invasive Aspergillosis (*n* = 103)	Mucormycosis (*n* = 62)	Others (*n* = 1)	Total (*n* = 253)
Pulmonary (%)	0	2 (5.6)	94 (91.3)	12 (19.3)	0	108 (42.7)
Sinus (%)	0	0	6 (5.8)	40 (64.5)	0	46 (18.2)
Blood (%)	53 (100)	0	0	0	0	53 (20.9)
Cerebral (%)	0	32 (94.1)	0	2 (3.2)	0	34 (13.4)
Others (%)	0	0	2 (1.9)	2 (3.2)	1 (100)	5 (2)
Disseminated (%)	0	0	1 (0.01)	6 (9.7)	0	7 (2.8)

**Table 2:** Demographic and clinical characteristics of IFI patients.

Variables	Total Patients (*n*= 253)	Proven IFI *n* = 134	Probable/Putative IFI *n* = 119	*p*-Value	Univariate OR (95% CI)	Multivariate OR (95% CI)
Age (years) Median, IQR (range)	40, 33 (0.06–87)	35, 34 (0.06–83)	43, 31 (1–87)	**0.003**	0.98 (0.97–0.99)	
Males, *n* (%)	167	86 (51.5)	81 (48.5)	0.51	0.84 (0.49–1.41)	
Hospitalization (days) Median, IQR (range)	19, 15 (1–171)	21, 15 (1–137)	17, 16 (1–171)	0.86	0.99 (0.98–1)	
General ward, *n* (%)	141	71 (50.3)	70 (49.6)	0.41		
High-efficiency particulate air-filtered room, *n* (%)	56	34 (60.7)	22 (39.3)		1.52 (0.81–2.86)	1.65 (0.8–3.4)
Intensive care unit (ICU), *n* (%)	56	29 (51.8)	27 (48.2)		1.05 (0.56–1.96)	1.2 (0.6–2.4)
Chronic granulomatous diseases, *n* (%)	50	23 (46)	27 (54)	0.34	0.7 (0.37–1.31)	
Long-term corticosteroids, *n* (%)	62	40 (64.5)	22 (35.5)	**0.04**	1.87 (1.03–3.39)	2.7 (1.34–5.45)
Diabetes mellitus, *n* (%)	60	36 (60)	24 (40)	0.2	1.45 (0.8–2.61)	1.3 (0.6–2.8)
Hematological malignancy, *n* (%)	50	12 (24)	38 (76)	**0.00**	0.2 (0.1–0.42)	
Other cancers, *n* (%)	8	4 (50)	4 (50)	1	0.88 (0.21–3.61)	
Acquired immunodeficiency syndrome, *n* (%)	14	10 (71.4)	4 (28.6)	0.16	2.31 (0.7–7.59)	4.6 (1.3–16.6)
Chronic liver disease, *n* (%)	40	33 (82.5)	7 (17.5)	**0.00**	5.22 (2.21–12.33)	6.9 (2.8–17.2)
Pulmonary manifestations, *n* (%)	78	18 (23.08)	60 (76.9)	**0.00**	0.15 (0.08–0.28)	
Chronic kidney disease, *n* (%)	87	40 (46)	47 (54)	0.11	0.65 (0.35–1.09)	
Coronary artery disease, *n* (%)	9	8 (89)	1 (11)	**0.03**	7.49 (0.92–60.8)	17 (2–142)
Multiorgan involvement, *n* (%)	8	8 (100)	0	**0.00**	1	
Trauma, *n* (%)	15	11 (73.3)	4 (26.7)	0.11	2.57 (0.79–8.3)	3.9 (1–14)
Antifungal administration days median, IQR (range)	14, 10 (2–138)	14, 7 (3–87)	21, 20 (2–138)	**0.00**	0.95 (0.93–0.97)	
Fungal etiology				**0.00**	0.31 (0.22–0.43)	
Candidemia, *n* (%)	53	53	0			
Cryptococcosis, *n* (%)	34	34	0			
Invasive aspergillosis, *n* (%)	103	14 (13.6)	89 (86.4)			
Mucormycosis, *n* (%)	62	33 (53.2)	29 (46.7)			
Taleromycosis, *n* (%)	1	0	1 (100)			
30-day outcome, *n* (%)				0.4	1.19 (0.72–1.96)	
Survived, *n* (%)	143	73 (51)	70 (49)			
Expired, *n* (%)	110	61 (55.4)	49 (44.5)			

Note: IFI, invasive fungal infection; *n*, total number of patients; IQR, inter quartile range; OR, odds ratio; CI, confidence interval.

**Table 3:** Multivariate analysis showing the interdependent variables as significant predictors of mortality in different IFI.

Variables	Invasive Candidiasis OR (95% CI)	Cryptococcosis OR (95% CI)	Invasive Aspergillosis OR (95% CI)	Mucormycosis OR (95% CI)
Age	1.07 (1.01–1.14)		1.07 (1.03–1.11)	1.01 (0.96–1.06)
Sex	3.27 (0.52–20.46)		1.39 (0.29–6.6)	
Hospitalization duration		1.04 (0.85–1.27)		
High-efficiency particulate air-filtered room	31.14 (1.72–560)	5.3 (0.75–37.38)		
Intensive care unit (ICU)	10.49 (0.99–110.3)		3.15 (0.84–11.75)	
Chronic granulomatous diseases		1.08 (0.09–12.55)		
Long-term corticosteroids			2.63 (0.6–11.9)	
Diabetes mellitus		3.77 (0.19–74.48)		
Hematological malignancy			15 (2.9–77.9)	
Acquired immunodeficiency syndrome		3.4 (0.23–48.94)		
Pulmonary manifestations	2.95 (0.33–26.06)			1.35 (0.4–4.6)
Chronic kidney disease		1.07 (0.15–7.42)	3.9 (1.1–13.7)	
Multiorgan involvement	9.5 (0.94–95.7)			
Mechanical ventilation			2.99 (0.76–11.63)	
Sepsis			5.8 (0.79–42.3)	
Symptom duration				1.08 (0.83–1.4)
Ketoacidosis				1.13 (0.12–10.68)
Radiological finding				
Lung collapse			1.5 (0.05–40.6)	
Nodules/ground glass opacities			1.5 (0.22–9.96)	100.5 (1.44–7006)
Consolidation				9.69 (0.09–997)
Sinus thickening				1.02 (0.13–7.57)
Galactomannan antigen index ≥1			2.72 (0.76–9.65)	
Species isolation				
	*Candida tropicalis*: 12.9 (1.11–150)			*Rhizopus microsporus*: 1.2 (0.09–15.5)
	*C. parapsilosis*: 1.2 (0.18–7.9)			*Mucor circinelloides*: 2.06 (0.01–328.9)
	*C. pelliculosa*: 1.2 (0.03–43.5)			

Note: IFI, invasive fungal infections; OR, odds ratio; CI, confidence interval.

**Table 4:** MIC range with geometric mean, mode, MIC_50_, and MIC_90_ values for the different fungal species from IFI cases by CLSI methodology.

Fungal Isolate	*N*	Amphotericin B		Fluconazole		Voriconazole		Itraconazole		Posaconazole		Caspofungin		Micafungin		Flucytosine	
		MIC_50_/MIC_90_; MIC Range	Mode, GM	MIC_50_/MIC_90_; MIC Range	Mode, GM	MIC_50_/MIC_90_; MIC Range	Mode, GM	MIC_50_/MIC_90_; MIC Range	Mode, GM	MIC_50_/MIC_90_; MIC Range	Mode, GM	MIC_50_/MIC_90_; MIC Range	Mode, GM	MIC_50_/MIC_90_; MIC Range	Mode, GM	MIC_50_/MIC_90_; MIC Range	Mode, GM
*Candida* sp.	53	0.25/0.5; 0.03–1	0.5, 0.217	0.5/8; 0.125–64	0.25, 0.812	0.03/0.25; 0.03–0.5	0.03, 0.063	0.06/0.5; 0.03–1	0.03, 0.081	0.03/0.5; 0.03–1	0.03, 0.054	0.125/0.5; 0.015–1	0.015, 0.097	0.015/0.5; 0.015–2	0.015, 0.047		
*Candida albicans*	14	0.06–0.5	0.5, 0.26	0.125–64	0.25, 0.82	0.03–0.5	0.03, 0.07	0.03–1	0.03, 0.11	0.03–0.5	0.03, 0.05	0.015–0.5	0.25, 0.08	0.015–1	0.015, 0.035		
*C. tropicalis*	10	0.03–0.5	0.03, 0.107	0.25–2	0.5, 0.466	0.03–0.125	0.03, 0.039	0.03–0.125	0.03, 0.045	0.03–0.5	0.03, 0.056	0.015–0.5	0.125, 0.107	0.015–1	0.015, 0.065		
*C. parapsilosis*	14	0.125–1	0.5, 0.304	0.125–64	0.5, 1.034	0.03–0.5	0.03, 0.063	0.03–1	0.03, 0.081	0.03–1	0.03, 0.054	0.015–1	0.25, 0.105	0.015–0.5	0.015, 0.042		
*C. guillermondii*	2	0.03–0.25		0.5–32		0.03–0.06		0.03–0.25		0.125–0.5		0.125–1		0.5–2			
*C. pelliculosa*	2	0.125–0.25		0.25–2		0.03		0.03		0.03		0.015–0.06		0.015–0.5			
*C. auris*	2	0.125		0.125–0.25		0.03		0.03		0.03		0.03–0.06		0.015			
*C. glabrata*	8	0.125–0.5	0.5, 0.272	0.25–8	1, 1	0.03–0.5	0.25, 0.123	0.06–0.5	0.06, 0.122	0.03–2	0.03, 0.06	0.015–1	0.125, 0.16	0.015–0.5	0.015, 0.072		
*Lodderomyces longisporus*	1	0.125		0.5		0.03		0.03		0.03		0.015		0.015			
*Cryptococcus neoformans*	24	0.25/1; 0.03–1	0.25, 0.342	2/4; 0.5–8	2, 2.181	0.03/0.06; 0.03–0.06	0.03, 0.033									2/2 0.25–4	2, 1.414
*Aspergillus* sp.	103	1/2; 0.03–4	1, 0.831			0.5/1; 0.03–2	0.5, 0.285	0.5/1; 0.03–2	0.5, 0.259	0.06/0.25; 0.03–0.25	0.03, 0.081	0.06/0.125; 0.015–1	0.015, 0.046	0.015/ 0.015; 0.015–0.03	0.015, 0.016		
*Aspergillus flavus*	42	2/4; 0.06–4	2, 1.559			0.5/1; 0.03–1	0.5, 0.363	0.25/1; 0.03–2	0.5, 0.255	0.06/0.25; 0.03–0.5	0.03, 0.085	0.06/0.125; 0.015–0.25	0.06, 0.045	0.015/0.015; 0.015–0.03	0.015, 0.016		
*A. fumigatus*	43	0.5/2; 0.03–2	1, 0.539			0.25/1; 0.03–1	0.5, 0.24	0.25/1; 0.03–2	0.5, 0.251	0.06/0.25; 0.03–0.5	0.03, 0.067	0.06/0.125; 0.015–1	0.015, 0.049	0.015/0.015; 0.015–0.03	0.015, 0.015		
*A. terreus*	7	0.5–1	1, 0.905			0.06–1	0.5, 0.272	0.125–0.5	0.5, 0.25	0.03–0.5	0.125, 0.124	0.03–0.25	0.06, 0.044	0.015–0.03	0.015, 0.016		
*A. nidulans*	3	0.06–1	1, 0.391			0.06–0.5	-, 0.155	0.03–0.5	0.5, 0.195	0.03–0.125	0.125, 0.077	0.03–0.125	-, 0.06	0.015–0.03	0.015, 0.018		
*A. niger*	8	0.06–2	0.25, 0.383			0.06–2	0.06, 0.268	0.06–2	0.5, 0.383	0.03–0.5	0.25, 0.134	0.015–0.25	0.015, 0.030	0.015	0.015, 0.015		
*Mucorales*	54	0.125/0.5; 0.06–1	0.06, 0.138					0.5/1; 0.06–1	0.5, 0.450	0.25/1; 0.03–2	0.25, 0.361						
*Rhizopus arrhizus*	30	0.125/0.25; 0.06–1	0.125, 0.135					0.5/1; 0.06–1	1, 0.499	0.25/2; 0.03–2	0.25, 0.475						
*R. microsporus*	13	0.06–0.5	0.06, 0.115					0.25–1	0.5, 0.5	0.03–1	1, 0.360						
*R. pusillus*	1	0.125						0.125		0.25							
*Lichtheimia corymbifera*	4	0.25	0.25, 0.25					0.125–0.25	0.25, 0.21	0.125	0.125, 0.125						
*L. ramosa*	1	0.25						0.5		0.25							
*Apophysomyces variabilis*	1	0.06						0.25		0.03							
*Mucor circinelloides*	3	0.25	0.25, 0.25					0.5–1	0.5, 0.629	0.25–0.5	0.25, 0.314						
*Conidiobolus coronatus*	1	0.06						0.25		0.25							
*Taleromyces marneffi*	1	0.125				0.06		0.125		0.03		0.015		0.015			

Note: *N*, total number of isolates; MIC, minimum inhibitory concentration; GM, geometric mean, -, not calculated.

**Table 5:** MIC interpretation for various fungi from IFI cases.

Fungal Isolate	*N*	Amphotericin B		Fluconazole		Voriconazole		Itraconazole		Posaconazole		Caspofungin		Micafungin		Flucytosine
		S	R	S	I/R	S	I/R	S	I/R	S	R	S	I/R	S	I/R	S
*Candida albicans*, *n* (%)	14	14 (100)	0	11 (78.6)	I: 2 (14.3); R: 1 (7.1)	11 (78.6)	I: 3 (21.4)	10 (71.4)	I: 3 (21.4); R: 1 (7.1)			13 (92.9)	I: 1 (7.1)	14 (100)	0	
*C. tropicalis*, *n* (%)	10	10 (100)	0	10 (100)	0	10 (100)	0	10 (100)	0			8 (80)	I: 2 (20)	7 (70)	I: 2 (20); R: 1 (10)	
*C. parapsilosis*, *n* (%)	14	14 (100)	0	10 (71.4)	I: 1 (7.1); R: 3 (21.4)	11 (78.6)	I: 3 (21.4)	10 (71.4)	I: 3 (21.4); R: 1 (7.1)			14 (100)	0	14 (100)	0	
*C. guillermondii*, *n* (%)	2	2 (100)	0	1 (50)	1 (50)	2 (100)	0	2 (100)	0			2 (100)	0		I: 1 (50); R: 1 (50)	
*C. pelliculosa*, *n* (%)	2	2 (100)	0	2 (100)	0	2 (100)	0	2 (100)	0			2 (100)	0	2 (100)	0	
*C. auris*, *n* (%)	2	2 (100)	0	2 (100)	0	2 (100)	0	2 (100)	0			2 (100)	0	2 (100)	0	
*C. glabrata*, *n* (%)	8	8 (100)	0	8 (100)	0	3 (37.5)	I: 5 (62.5)	5 (62.5)	I: 3 (37.5)			4 (50)	I: 1 (12.5); R:3 (37.5)	4 (50)	I: 1 (12.5); R: 3 (37.5)	
*Lodderomyces longisporus*, *n* (%)	1	1 (100)	0	1 (100)	0	1 (100)	0	1 (100)	0			1 (100)	0	1 (100)	0	
*Cryptococcus neoformans*, *n* (%)	24	24 (100)	0	24 (100)	0	24 (100)	0									24 (100)
*Aspergillus flavus*, *n* (%)	42	42 (100)	0			42 (100)	0	41 (97.6)	1 (2.3)	42 (100)	0	42 (100)	0	42 (100)	0	
*A. fumigatus*, *n* (%)	43	43 (100)	0			43 (100)	0	42 (97.6)	1 (2.3)	43 (100)	0	41	2 (4.7)	43 (100)	0	
*A. terreus*, *n* (%)	7	7 (100)	0			7 (100)	0	7 (100)	0	7 (100)	0	7 (100)	0	7 (100)	0	
*A. nidulans*, *n* (%)	3	3 (100)	0			3 (100)	0	3 (100)	0	3 (100)	0	3 (100)	0	3 (100)	0	
*A. niger*, *n* (%)	8	8 (100)	0			8 (100)	0	7 (87.5)	1 (12.5)	8 (100)	0	8 (100)	0	8 (100)	0	
*Rhizopus arrhizus*, *n* (%)	54	54 (100)	0			54 (100)	0	42 (77.7)	12 (22.2)	39 (72.2)	15 (27.8)					
*R. microsporus*, *n* (%)	30	30 (100)	0			30 (100)	0	27 (90)	3 (10)	23 (76.6)	7 (23.3)					
*R. pusillus*, *n* (%)	13	13 (100)	0			13 (100)	0	13 (100)	0	13 (100)	0					
*Lichtheimia corymbifera*, *n* (%)	1	1 (100)	0			1 (100)	0	1 (100)	0	1 (100)	0					
*L. ramosa*, *n* (%)	4	4 (100)	0			4 (100)	0	4 (100)	0	4 (100)	0					
*Apophysomyces variabilis*, *n* (%)	1	1 (100)	0			1 (100)	0	1 (100)	0	1 (100)	0					
*Mucor circinelloides*, *n* (%)	1	1 (100)	0			1 (100)	0	0	1 (100)	1 (100)	0					
*Conidiobolus coronatus*, *n* (%)	3	3 (100)	0			3 (100)	0	3 (100)	0	3 (100)	0					
*Taleromyces marneffi*, *n* (%)	1	1 (100)	0			1 (100)	0	1 (100)	0	1 (100)	0					

Note: *N*, total number of isolates; S, susceptible; I, intermediate; R, resistant.

## Supplementary Materials

The following is available online at https://www.mdpi.com/article/10.3390/jof8010033/s1, Figure S1: Fungal species distribution in samples from IFI cases (*n* = 253).
