# Supplementary material for: Epidemiology and Antifungal Susceptibility Patterns of Invasive Fungal Infections (IFIs) in India: A Prospective Observational Study
Source: J Fungi (Basel). 2021 Dec 30;8(1):33. doi: 10.3390/jof8010033 (PMC8777790; doi:10.3390/jof8010033)
Supplement: Supplementary file 1 [file jof-08-00033-s001.zip › jof-1464005-supplementary.pdf]

Supplementary Figure S1: Fungal species distribution in samples from IFI cases (n = 253)

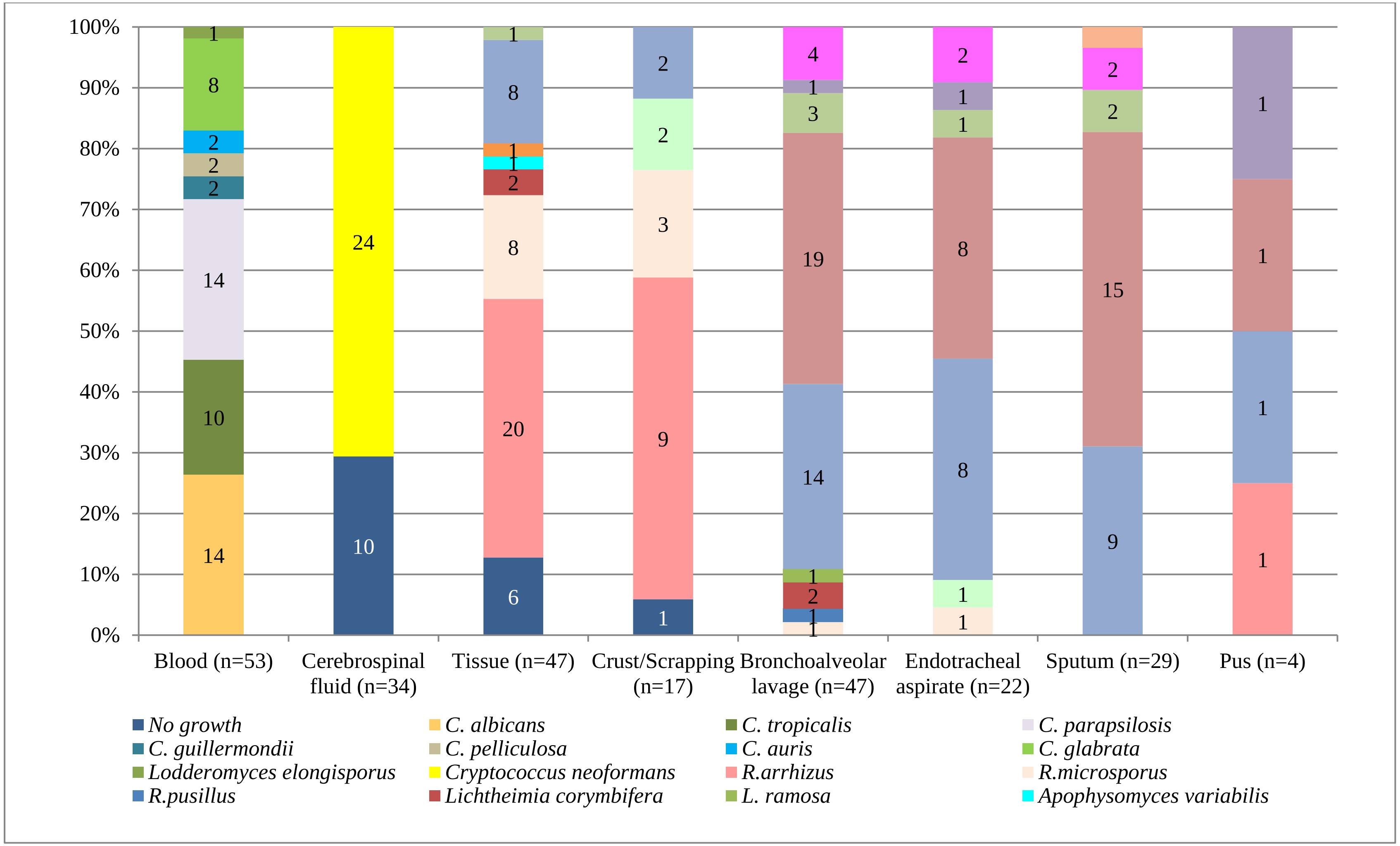

Note: IFI: invasive fungal infections
